# Potential impacts of the 2017 American College of Cardiology/American Heart Association high blood pressure guideline on Chinese adults and how to address them

**DOI:** 10.1186/s12872-020-01523-z

**Published:** 2020-05-19

**Authors:** Yundi Jiao, Zhaoqing Sun, Yanxia Xie, Jia Zheng, Zhao Li, Xiaofan Guo, Yue Dai, Liqiang Zheng, Yingxian Sun

**Affiliations:** 1 Department of Cardiology, Shengjing Hospital of China Medical University, 36 Sanhao Street, Heping District, Shenyang, 110004 People’s Republic of China; 2 Department of Clinical Epidemiology, Department of Library, Shengjing Hospital of China Medical University, 36 Sanhao Street, Heping District, Shenyang, 110004 People’s Republic of China; 3 Department of Cardiology, the First Affiliated Hospital of China Medical University, Shenyang, 110001 People’s Republic of China

**Keywords:** Hypertension, Guideline, Prevalence, Antihypertensive medications

## Abstract

**Background:**

The current analysis was performed to estimate the percentage and number of Chinese adults with hypertension and the percentage and number of Chinese adults recommended to receive pharmacological antihypertensive treatment according to the 2017 American College of Cardiology/American Heart Association (ACC/AHA) guideline compared with the same parameters according to the 2010 Chinese guideline.

**Methods:**

We used 2011 data from the China Health and Nutrition Survey (CHNS). A total of 12,499 Chinese adults aged ≥18 years with complete blood pressure (BP) values were selected for the present analysis.

**Results:**

The crude prevalence rates (95% CI) of hypertension according to the definitions from the 2017 ACC/AHA guideline and the 2010 Chinese guideline were 58.0% (57.2 to 58.9%) and 25.4% (24.7 to 26.2%), respectively. Moreover, the percentage of the participants recommended to take antihypertensive medications were 31.5 and 28.8%, respectively. Among adults who took antihypertensive medications, 88.8% had above-goal BP levels compared to 53.3%. Overall, 613.3 million Chinese adults (aged ≥18 years) met the criteria for hypertension according to the 2017 ACC/AHA guideline, and 267.7 million met the criteria according to 2010 Chinese guideline. An additional 28.4 million (2.7%) Chinese adults were recommended to take antihypertensive medication.

**Conclusions:**

The present analysis revealed that the 2017 ACC/AHA hypertension guideline will result in a substantial increase in the percentage and number of Chinese adults defined as having hypertension and a small increase in the percentage of adults who are recommended to take antihypertensive medications compared to the same parameters based on the 2010 Chinese guideline. More intensive management and antihypertensive medications use are suggested to improve the control rate of hypertension among Chinese adults.

## Backgroud

On November 13, 2017, the American College of Cardiology/American Heart Association (ACC/AHA) Guideline for the Prevention, Detection, Evaluation and Management of High Blood Pressure in Adults was published [1]. The guideline substantially updated the Seventh Report of the Joint National Committee on the Prevention, Detection, Evaluation and Treatment of High Blood Pressure (JNC7) in 2003 [2] and provided more information on the prevention and treatment of hypertension. The guideline will help us provide more intensive care for high blood pressure and reduce the incidence of cardiovascular diseases such as stroke, heart attack, and heart failure. Paul Muntner et al. [3] indicated that the 2017 ACC/AHA guideline will increase the prevalence of hypertension, compared to that based on the JNC7 guideline, from 31.9 to 45.6% based on the representative sample of the 2011–2014 National Health and Nutrition Examination Survey (NHANES). However, the percentage of US adults who were recommended to take antihypertensive medication increased slightly by 1.9% (from 34.3 to 36.2%). In addition, among US adults taking antihypertensive medication, 53.4 and 39.0% had blood pressure above the treatment goal according to the 2017 ACC/AHA and JNC7 guideline, respectively. A substantial proportion of US adults taking antihypertensive medication are recommended to undergo more intensive blood pressure lowering based on the 2017 ACC/AHA guideline.

China is experiencing a high prevalence and a low rate of treatment and control of hypertension [4, 5]. In recent decades, the prevalence and incidence of hypertension have increased markedly in China, especially in rural areas [4, 6, 7]. Recently, a study that included 1.7 million adults (aged 35 to 75 years) revealed that the prevalence of hypertension was 44.7%, whereas the rates of hypertensive medication used and BP goal achievement in hypertensive patients were 30.1 and 7.2% [5], respectively. Compared to the JNC7 guideline and the Chinese guideline for high blood pressure, the 2017 ACC/AHA guideline recommends using lower systolic blood pressure (SBP) and diastolic blood pressure (DBP) levels to define hypertension (130/80 mmHg). The new definition has substantially increased the number of hypertensive patients in the US. The potential impact of the 2017 ACC/AHA guideline in China remains to be elucidated.

The current analysis used data from the China Health and Nutrition Survey (CHNS) in 2011 to estimate the percentage and number of Chinese adults with hypertension and those recommended to undergo pharmacological antihypertensive treatment according to the 2017 ACC/AHA guideline compared with the same parameters based on the 2010 Chinese guideline for the Management of Hypertension. In addition, we estimated the percentage and number of Chinese adults taking antihypertensive medication with blood pressure above the goal using the targets from each guideline.

## Methods

### Study sample

The CHNS is a follow-up survey regarding nutrition and food safety conducted by the Chinese Center for Disease Control and Prevention in collaboration with the Population Center of the University of North Carolina in the United States. The CHNS aimed to develop a longitudinal and multipurpose survey that could help the group measure health factors of interest, such as sociological, economic and demographic factors, for use by the CAPM (formerly the Chinese Academy of Preventive Medicine) and scholars [8]. Since 1989, the CHNS has been conducted nine times (1989, 1991, 1993, 1997, 2000, 2004, 2006, 2009, and 2011) and has covered nine provinces (Liaoning, Heilongjiang, Jiangsu, Shandong, Henan, Hubei, Hunan, Guangxi and Guizhou), including urban and rural areas categorized by income (low, middle, and high). CHNS used a multistage stratified cluster random sampling method, and a weighted sampling scheme was used to randomly select four counties in each province [9]. We used the 2011 health data of residents for this analysis. For the present analysis, the 2011 data were restricted to those of adult participants aged ≥18 years (*n* = 13,052). Participants were excluded if the three blood pressure (SBP or DBP) measurements were not available in the survey (*n* = 553), leaving 12,499 Chinese adults aged ≥18 years with complete blood pressure values for the present analysis.

### Data collection

In the CHNS, standard questionnaires were used to collect basic information and related biochemical indicators were measured in a national central lab in Beijing (medical laboratory accreditation certificate ISO 15189: 2007) with strict quality control [10]. Blood pressure was measured by trained examiners using a mercury sphygmomanometer with a suitable cuff size according to a standard protocol [5]. Three measurements were taken 10 min after rest, and the average of the three measurements was used for the analysis [11]. In addition, the questionnaire asked whether the participant had a history of use of antihypertensive drugs.

Cardiovascular disease (CVD) history was defined by a self-reported previous diagnosis of myocardial infarction or stroke. The definitions of hypertension, recommended antihypertensive drug standards and recommended blood pressure targets for adults taking antihypertensive drugs referred to the 2017 ACC/AHA guideline and the 2010 Chinese guideline for the management of hypertension are presented in Table 1.

**Table 1:** Blood pressure levels used to define hypertension, recommend antihypertensive medication, and treatment goal according to the 2017 ACC/AHA Guideline and the 2010 Chinese Guideline

	Guideline – Definition of hypertension	
	2017 ACC/AHA	2010 Chinese
SBP, mmHg	≥130	≥140
DBP, mmHg	≥80	≥90
	Guideline – Recommended antihypertensive medication	
	2017 ACC/AHA	2010 Chinese
SBP, mmHg		
General population	≥140	≥140
Aged ≥65 years	≥130	≥140
Diabetes	≥130	≥130
CHD or stroke	≥130	≥140
DBP, mmHg		
General population	≥90	≥90
Aged ≥65 years	≥80	≥90
Diabetes	≥80	≥80
CHD or stroke	≥80	≥90
	Treatment goal among those taking antihypertensive medication	
	2017 ACC/AHA	2010 Chinese
SBP, mmHg		
General population	< 130	< 140
Aged ≥65 years	< 130	< 150
Diabetes or CHD or stroke	< 130	< 130
DBP, mmHg		
General population	< 80	< 90
Diabetes or CHD or stroke	< 80	< 80

*SBP* systolic blood pressure; *DBP* diastolic blood pressure; *CHD* coronary heart disease

### Statistical analysis

Continuous variables were presented as mean ± standard deviation (SD) or median [interquartile range, IQR], while categorical variables were expressed as counts and percentages (%). We calculated the distribution of the Chinese adults across five groups, including four groups that did not take antihypertensive medication (SBP/DBP < 120/< 80, 120–129/< 80, 130–139/80–89, and ≥ 140 / 90 mmHg) and a group taking antihypertensive drugs. Patient groups were compared by *x*^2^ tests for categorical variables or one-way analysis of variance for continuous variables. We calculated the percentage and number (95% CI) of adults with hypertension in China and the percentage and number of people who were recommended to receive antihypertensive therapy based on the 2017 ACC/AHA guideline, the 2010 Chinese guideline, and the differences between the two guidelines (the 2017 ACC/AHA guideline but not the 2010 Chinese guideline). These calculations were performed in the general population and in different subgroups (such as groups with different ages and sexes). Moreover, the above method was used to calculate the Chinese demographic and clinical characteristics of blood pressure above the goal according to the 2017 ACC/AHA guideline and the 2010 Chinese guideline. Data from the sixth national census in 2010 were used to calculate the numbers of individuals with prevalent hypertension, who are recommended to take antihypertensive medications, and who have blood pressure above the goal. All analyses were performed with SPSS statistical software version 13.0 (SPSS Inc., Chicago, IL, USA) and SAS statistical software version 9.2 (SAS Institute Inc., Carey, NC, USA). A *P* value less than 0.05 was accepted as indicating statistical significance.

## Results

The median age of the present study participants was 51.0 ± 15.2 years, and 53.3% were women. A total of 13.4% (1663) of Chinese adults were taking antihypertensive medications. A total of 32.0, 9.7, 30.1 and 14.6% of Chinese adults not taking antihypertensive medications had SBP/DBP levels of < 120/80, 120–129/< 80, 130–139/80–89, and ≥ 140/90 mmHg, respectively (Table 2). Table 2 shows the baseline characteristics of the study participants according to different BP subgroups. As expected, Chinese adults with higher blood pressure were older and more likely to be men, current smokers and to have diabetes and a history of stroke and coronary heart disease (all *P* < 0.001).

**Table 2:** Characteristics of Chinese adults (aged ≥18 years) by blood pressure levels and antihypertensive medication use based on the 2011 China Health and Nutrition Survey (*n* = 12,499)

Characteristics	SBP/DBP categories in mmHg among Chinese adults not taking antihypertensive medication				Taking antihypertensive medication (*n* = 1663)	*P*-Value^b^
	<120/80 (*n* = 4027)	120–129/<80 (*n* = 1218)	130–139/80–89 (*n* = 3768)	≥140/90 (*n* = 1823)		
Percentage of Chinese population	32.2	9.7	30.1	14.6	13.4	<0.001
Population characteristics ^a^						
Age, year	43.0(33.0–55.0)	51.0(39.0–61.0)	50.0(41.0–59.0)	57.0(48.0–66.0)	62.0(56.0–71.0)	<0.001
Women, %	63.9	48.9	46.8	44.2	53.3	<0.001
Current smoking, %	22.9	34.8	34.3	38.3	30.5	<0.001
Diabetes, %	1.4	2.9	3.0	4.4	4.1	<0.001
SBP, mmHg	108.1 ± 8.1	123.2 ± 3.1	124.5 ± 8.3	144.6 ± 15.3	124.5 ± 17.7	<0.001
DBP, mmHg	70.0 ± 6.2	74.0 ± 4.8	81.8 ± 4.2	90.9 ± 10.0	79.3 ± 10.7	<0.001
The history of coronary heart disease, %	0.4	0.4	0.5	0.7	0.9	<0.001
The history of Stroke, %	0.4	0.9	0.6	1.4	1.6	<0.001
The history of CVD^c^, %	0.7	1.2	1.0	2.0	2.4	<0.001

^a^ Population characteristics in the table are percentage or mean (standard deviation)

^b^*P*-values were calculated by one-way ANOVA or chi-square test to compare differences within different blood pressure categories

^c^Defined by a self-reported history of stroke or coronary heart disease

2017 ACC/AHA Guideline - 2017 American College of Cardiology / American Heart Association Guideline for the Prevention, Detection, Evaluation and Management of High Blood Pressure in Adults

*SBP* systolic blood pressure; *DBP* diastolic blood pressure; *CVD* cardiovascular disease

The prevalence rates (95% CI) of hypertension according to the definitions from the 2017 ACC/AHA guideline and the 2010 Chinese guideline were 58.0% (57.2 to 58.9%) and 25.4% (24.7 to 26.2%), respectively (Table 3). The prevalence of hypertension was higher when based on the 2017 ACC/AHA guideline compared to that based on the 2010 Chinese guideline within all age, sex, and CVD history subgroups. In addition, the difference in prevalence defined by the 2017 ACC/AHA guideline but not the 2010 Chinese guideline was significant among the different subgroups (*P* < 0.05) (Table 3).

**Table 3:** Prevalence (95% CI) of hypertension according to the definition from 2017 ACC/AHA Guideline and the 2010 China Hypertension Guideline based on the 2011 China Health and Nutrition Survey (*n* = 12,499)

	2017 ACC/AHA Guideline	2010 Chinese Hypertension Guideline	Difference (2017 ACC/AHA but not 2010 Chinese Hypertension Guideline)
Overall	58.0 (57.2, 58.9)	25.4 (24.7, 26.2)	32.6 (31.8, 33.4)
Age, years			
18–34	30.3 (28.3, 32.4)	3.4 (2.6, 4.2)	26.9 (25.0, 28.9)
35–44	47.2 (45.2,49.3)	10.4 (9.2, 11.7)	36.8 (34.8, 38.8)
45–54	61.1 (59.3, 62.9)	22.9 (21.3, 24.4)	38.2 (36.5, 40.0)
55–64	68.3 (66.6, 69.9)	35.5 (33.8, 37.2)	32.8 (31.1, 34.4)
65–74	72.9 (70.7, 75.1)	46.6 (44.1, 49.0)	26.3 (24.2, 28.5)
≥ 75	78.0 (75.1, 80.8)	51.4 (47.9, 54.8)	26.6(23.6, 29.7)^b^
Sex			
Men	64.4 (63.2, 65.6)	27.2 (26.1, 28.4)	37.2 (35.6, 38.4)
Women	52.5 (51.3, 53.7)	23.8 (22.8, 24.9)	28.6(22.5, 29.7)^b^
The history of CVD ^a^			
Yes	85.1 (81.1, 89.2)	71.3 (66.2, 76.4)	13.9 (10.0, 17.8)
No	57.4 (56.5, 58.2)	24.3 (23.5, 25.0)	33.1(32.2, 33.9)^b^

^a^Defined by a self-reported history of stroke or coronary heart disease

^b^Chi-square test for Comparison among different subgroups, *P* < 0.05

2017 ACC/AHA Guideline - 2017 American College of Cardiology / American Heart Association Guideline for the Prevention, Detection, Evaluation and Management of High Blood Pressure in Adults

The percentage of individuals recommended to take antihypertensive medications among Chinese adults was 31.5 and 28.8% according to the 2017 ACC/AHA guideline and 2010 Chinese guideline, respectively. An increase in the percentage of the population recommended to take antihypertensive medication based on the 2017 ACC/AHA guideline compared to that based on the 2010 Chinese guideline was present in all subgroups. The subgroups of individuals with an older age and a history of CVD had a relatively higher increase relative to adults with a young age and without CVD history (Table 4). Among Chinese adults with SBP/DBP of 130–139/80–89 mmHg, 12.0% were recommended to take antihypertensive medication according to the 2017 ACC/AHA guideline because they had diabetes, CVD history or SBP of 130–139 mmHg and were ≥ 65 years of age.

**Table 4:** Percentage (95% CI) of Chinese adults meeting the definition for recommended antihypertensive medication according to the 2017 ACC/AHA Guideline and the 2010 China Hypertension Guideline based on the 2011 China Health and Nutrition Survey (*n* = 12,499)

	Recommended antihypertensive medication		Difference (2017 ACC/AHA but not 2010 Chinese Hypertension Guideline)
	2017 ACC/AHA Guideline	2010 Chinese Hypertension Guideline	
Overall	31.5(30.7,32.3)	28.8(28.0,29.6)	2.7(2.4,3.0)
Age, years			
18–34	5.0(4.0,5.9)	5.0(4.0,5.9)	0.0(0.0,0.0)
35–44	12.4(11.1,13.8)	12.3(11.0,13.7)	0.1(0.03,0.2)
45–54	27.2(25.6,28.9)	27.2(25.6,28.8)	0.0(0.0,0.0)
55–64	40.1(38.4,41.9)	39.6(37.9,41.4)	0.5(0.2,0.7)
65–74	64.3(62.0,66.7)	50.9(48.5,58.4)	13.4(11.7,15.1)
≥ 75	68.8(65.6,72.0)	52.2(51.8,58.6)	13.6 (11.2,16.0)^b^
Sex			
Men	33.9(32.7,35.2)	31.1(29.9,32.3)	2.8(2.4,3.3)
Women	29.4(28.3,30.5)	26.8(25.7,27.8)	2.6(2.2,3.0)
The history of CVD ^a^			
Yes	85.1(81.1,89.2)	73.9(69.0,78.9)	11.2(7.7,14.8)
No	30.2(29.4,31.0)	27.7(26.9,28.5)	2.5 (2.2,2.8)^b^

^a^Defined by a self-reported history of stroke or coronary heart disease

^b^Chi-square test for Comparison among different subgroups, *P* < 0.05

2017 ACC/AHA Guideline - 2017 American College of Cardiology / American Heart Association Guideline for the Prevention, Detection, Evaluation and Management of High Blood Pressure in Adults

In 2011, 613.3 million Chinese adults (≥ 18 years) met the criteria for hypertension according to the 2017 ACC/AHA guideline compared with 267.7 million Chinese adults according to the 2010 Chinese guideline (Table 5). Using the recommendation from the 2017 ACC/AHA guideline, 332.0 million Chinese adults not taking antihypertensive medications met the criteria for treatment with antihypertensive medication in addition to nonpharmacological interventions, whereas 303.6 million met the criteria for treatment with nonpharmacological therapy. An additional 28.4 million Chinese adults were recommended to take antihypertensive medication according to the 2017 ACC/AHA guideline compared with the 2010 Chinese guideline.

**Table 5:** Number of China adults, in million, meeting the definition for hypertension and the definition for treatment with antihypertensive medication according to the 2017 ACC/AHA Guideline and the 2010 China Hypertension Guideline based on the 2011 China Health and Nutrition Survey

	2017 ACC/AHA Guideline		2010 Chinese Hypertension Guideline		Difference (2017ACC/AHA vs 2010 Chinese)	
	Hypertension	Recommended antihypertensive medication	Hypertension	Recommended antihypertensive medication	Hypertension	Recommended antihypertensive medication
Overall	611.3 (602.9, 620.8)	332.0 (323.6, 340.4)	267.7 (260.3, 276.1)	303.6 (295.1, 312.0)	343.6 (335.2, 352.0)	28.4 (25.3, 31.6)
Age, years						
18–34	50.1 (48.9, 55.0)	8.3 (6.6, 10.0)	5.6 (5.4, 8.0)	8.3 (6.1, 9.1)	44.5 (41.23, 48.1)	0.0 (0.0, 0.0)
35–44	91.1 (85.6, 97.8)	24.0(19.9, 26.6)	20.1 (18.7,21.4)	23.9 (21.3, 27.3)	71.1(65.3, 75.6)	0.2 (0.06, 0.40)
45–54	145.5 (140.6, 152.8)	64.7(59.8, 69.7)	54.3 (50.9, 58.9)	64.7 (60.7, 69.8)	91.2 (85.9, 96.2)	0.1 (0.08, 0.25)
55–64	173.6 (165.0, 177.3)	102.3(96.3, 106.2)	90.5 (85.7, 93.7)	101.1 (97.2, 106.3)	83.1 (79.0, 89.3)	1.2 (0.6, 2.0)
65–74	97.2 (91.7, 103.9)	85.9 (83.0, 89.6)	62.1 (58.9, 66.9)	68.0 (63.8, 72.9)	35.1 (30.9, 37.8)	17.8 (16.3, 19.2)
≥ 75	53.2 (48.9, 55.0)	46.8 (43.2, 49.8)	35.1 (32.1, 37.5)	37.6 (33.4, 39.5)	18.1(17.2, 20.6)	9.2 (7.8, 10.6)
Sex						
Men	316.7 (311.8, 324.0)	167.0 (162.7, 172.6)	133.9 (128.5, 139.2)	153.0(148.8, 157.8)	182.8 (178.7, 189.0)	13.9 (12.4, 15.4)
Women	294.6 (287.3, 299.5)	165.0(159.4, 169.3)	133.9 (128.5, 139.2)	150.6(145.7, 154.8)	160.7 (154.6, 164.9)	14.5 (13.1, 16.1)
The history of CVD^a^						
Yes	22.0 (18.3, 24.5)	21.9(19.9, 23.24)	18.2 (16.1, 21.4)	18.8(18.2, 21.3)	3.8 (2.4, 4.5)	2.7 (1.9, 3.8)
No	589.3 (586.8, 593.0)	310.1(308.8, 312.1)	249.5 (246.3, 251.6)	284.7(282.3, 288.4)	339.8 (338.8, 340.8)	25.5 (24.7, 26.6)

^a^Defined by a self-reported history of stroke or coronary heart disease

2017 ACC/AHA Guideline - 2017 American College of Cardiology / American Heart Association Guideline for the Prevention, Detection, Evaluation and Management of High Blood Pressure in Adults

Among these Chinese adults taking antihypertensive medications, 88.8% had above-goal BP according to the 2017 ACC/AHA guideline compared to 53.3% with above-goal BP according to the 2010 Chinese guideline (Table 6). Additionally, the prevalence of blood pressure above the goal, defined by the 2017 ACC/AHA guideline compared to that defined by the 2010 Chinese guideline, was more than 25% higher in each subgroup investigated except for those aged 45–54 years.

**Table 6:** Percentage (95% CI) of Chinese adults taking antihypertensive medication with blood pressure above goal according to the 2017 ACC/AHA Guideline and the 2010 China Hypertension Guideline based on the 2011 China Health and Nutrition Survey

	Blood pressure above goal according to:		Difference (2017 ACC/AHA but not 2010 Chinese Hypertension Guideline)
	2017 ACC/AHA Guideline	2010 Chinese Hypertension Guideline	
Overall	88.8 (87.2, 90.3)	53.3 (51.0, 55.7)	35.5 (33.1, 37.7)
Age, years			
18–34^c^	–	–	–
35–44	96.9 (92.6, 100.0)	71.9 (60.9, 82.9)	25.0 (14.4, 35.6)
45–54	90.1 (86.7, 93.5)	67.3 (62.0, 72.6)	22.8 (18.1, 27.5)
55–64	87.8 (85.1, 90.4)	62.1 (58.2, 66.0)	25.6 (22.1, 29.2)
65–74	88.6 (85.7, 91.6)	36.1 (31.6, 40.6)	52.5 (47.8, 57.2)
≥ 75	87.5 (83.6, 91.5)	41.5 (35.6, 47.4)	46.0 (40.0, 52.0)^b^
Sex			
Men	89.6 (87.3, 91.8)	53.2 (49.6, 56.8)	36.4 (32.9, 39.8)
Women	88.1 (86.0, 90.2)	53.5 (50.2, 56.7)	34.7 (31.6, 37.7)
The history of CVD^a^			
Yes	88.7 (84.2, 93.9)	48.4 (41.2, 55.6)	40.3 (33.3, 47.4)
No	88.8 (87.2, 90.4)	54.0 (51.4, 56.5)	34.8 (32.4, 37.2)

^a^ Defined by a self-reported history of stroke or coronary heart disease

^b^Chi-squaretest for Comparison among different subgroups, *P* < 0.05

^c^Small sample size (*n* = 2) is not adapted to calculate the percentage

2017 ACC/AHA Guideline - 2017 American College of Cardiology / American Heart Association Guideline for the Prevention, Detection, Evaluation and Management of High Blood Pressure in Adults

## Discussion

**Fig. 1:**
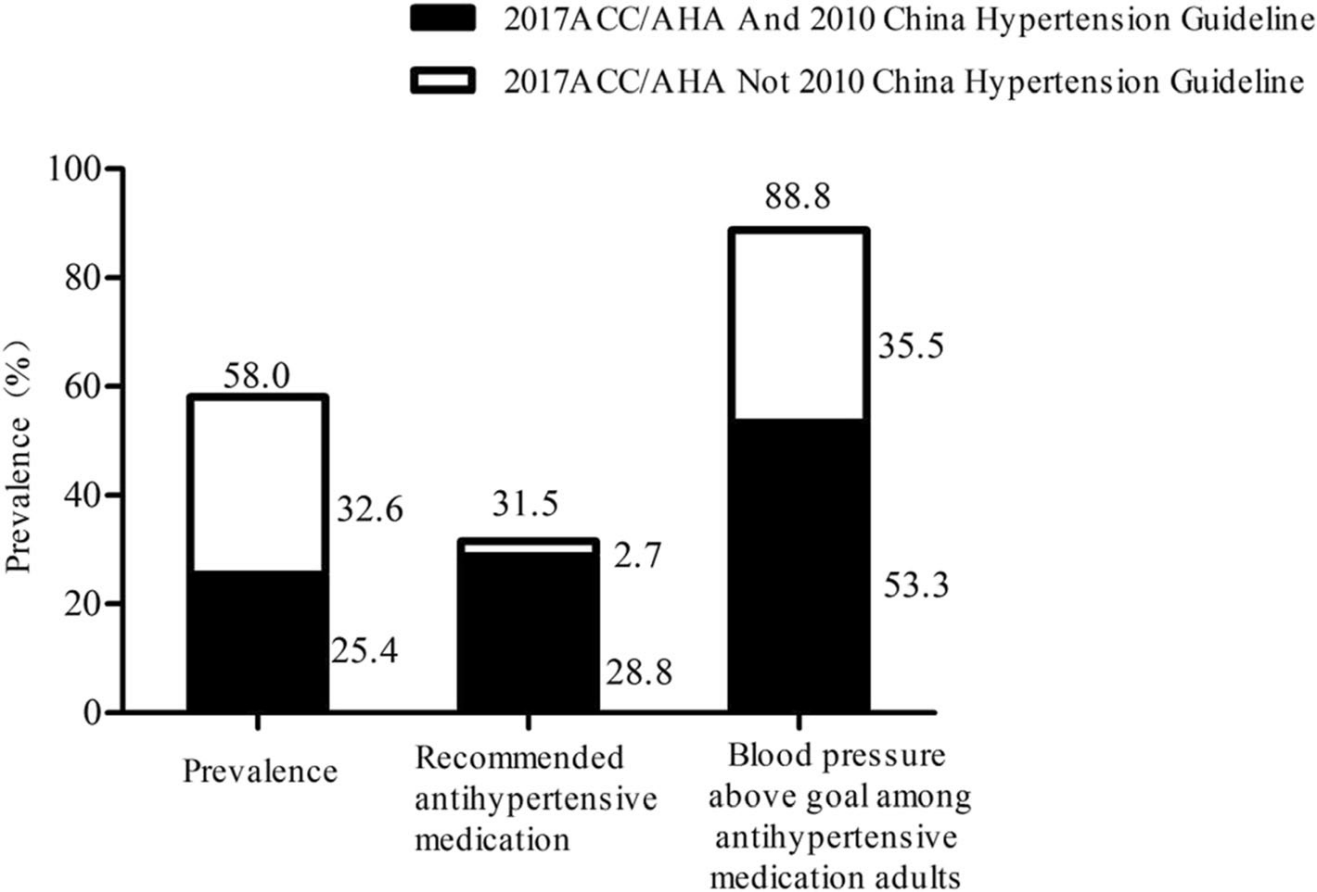
Prevalence of hypertension, recommendation for pharmacologic antihypertensive treatment, and blood pressure above goal among Chinese adults according to the 2017 ACC/AHA Guideline and the 2010 China Hypertension Guideline

**Fig. 2:**
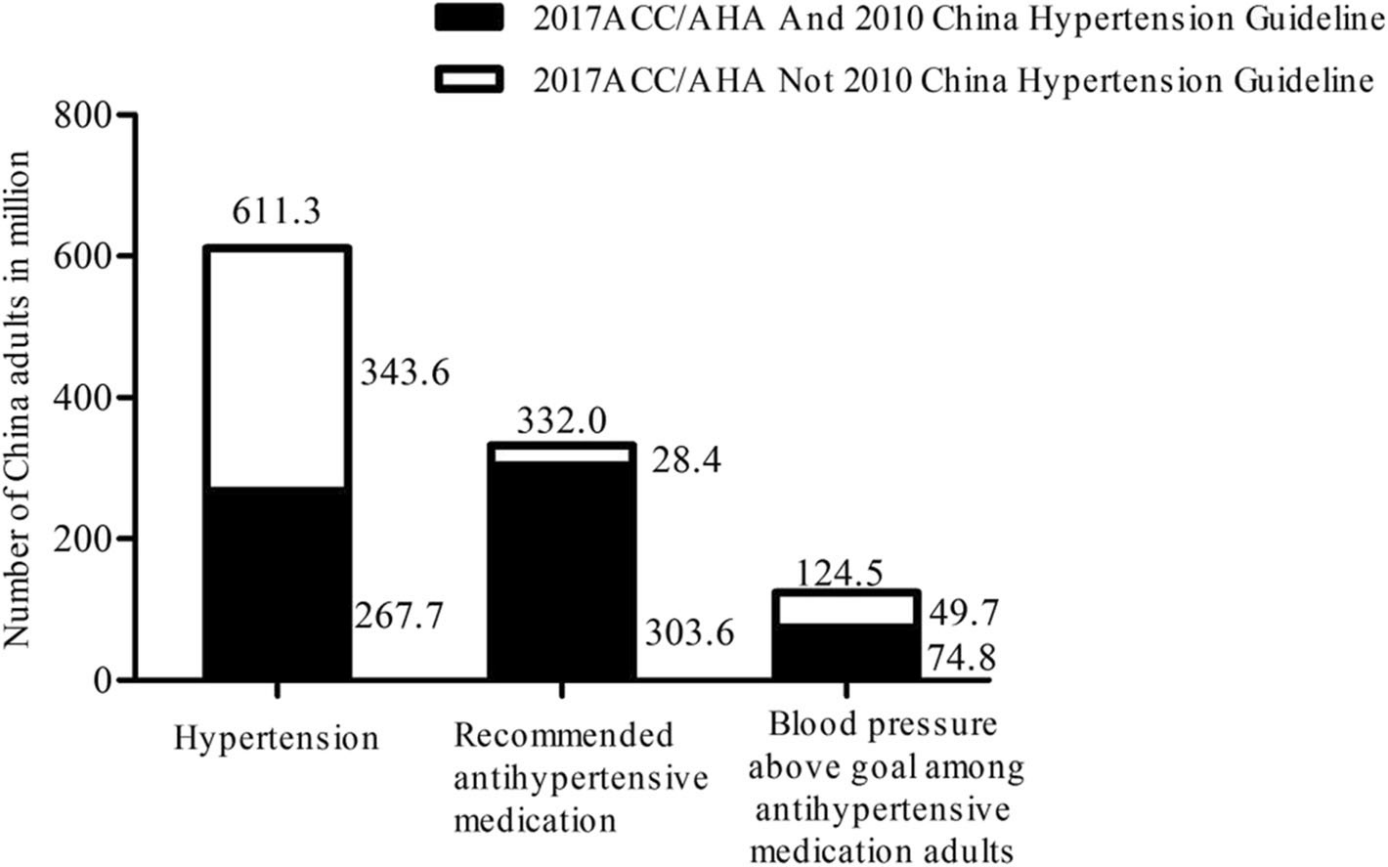
Numbers of hypertension, recommendation for pharmacologic antihypertensive treatment, and blood pressure above goal among Chinese adults according to the 2017 ACC/AHA Guideline and the 2010 China Hypertension Guideline

The present analysis indicates the potential impacts of the 2017 ACC/AHA guideline on the definition of hypertension, recommendation for antihypertensive medication in addition to nonpharmacological interventions and blood pressure goals in individuals receiving antihypertensive drug treatment among Chinese adults (Figs. 1 and 2). The present analysis revealed that the 2017 ACC/AHA hypertension guideline will result in a substantial increase in the percentage and number of Chinese adults defined as having hypertension. However, the percentage of adults who are recommended to take antihypertensive medications will only increase minimally (2.7%) according to the 2017 ACC/AHA guideline compared to that based on the 2010 Chinese guideline. In addition, 35.5% of Chinese adults taking antihypertensive medication had a blood pressure above the goal defined by the 2017 ACC/AHA guideline, whereas they would have met the BP goal according to the 2010 Chinese guideline. More intensive management and antihypertensive medications are suggested to improve the control rate of hypertension among Chinese adults.

There is no doubt that the percentage and number of individuals with hypertension will increase substantially with the change in definition of hypertension according to the 2017 ACC/AHA guideline [1]. The prevalence of hypertension among US adults will have a relative 40% increase (from 31.9 to 45.6%) [3]. Our study offers the exact data on the number of Chinese adults met the criteria for hypertension. The prevalence of hypertension among Chinese adults will increase from 25.6 to 55.6% according to the 2017 ACC/AHA guideline, and the number of individuals with hypertension will also increase steeply from 260 million to 630 million. Therefore, many new prevalent cases will be diagnosed because of the 2017 ACC/AHA guideline. An important question is how to face the challenge. In fact, the percentage of adults with SBP/DBP of 130–139/80–89 mmHg contributes to the deviation based on the 2017 ACC/AHA guideline. Although several observational studies have demonstrated an association between blood pressure of 130–139/80–89 mmHg and the risk of incident CVD [11–16], the 2017 ACC/AHA writing committee still emphasized that there is insufficient evidence to support a recommendation for antihypertensive drug treatment for these individuals to obtain more benefits [3]. However, the findings can provide an opportunity to discuss the value of nonpharmacological therapy in lowering blood pressure, to implement recommended lifestyle changes and to emphasize that blood pressure is a risk factor that can be controlled. Therefore, the aim of the definition is to raise the awareness of these individuals and to encourage the implementation of recommended lifestyle changes; the definition of hypertension is mostly acceptable in China.

In addition, according to the 2017 ACC/AHA guideline, the prevalence of recommended antihypertensive medication among US adults increased by 1.9% [3], while in the present study, the prevalence of recommended antihypertensive medication increased from 28.8 to 31.5% according to the 2017 ACC/AHA guideline compared to the 2010 Chinese guideline. The corresponding number of patients receiving antihypertensive medication will increase by 28.4 million. However, we have to consider that we did not consider adults with CVD risk > 10% because of the shortage of data in this study. Therefore, the percentage and number of individuals recommended to take antihypertensive medications will continue to increase if CVD risk is taken into account. The 2017 ACC/AHA guideline used a combination of CVD risk and blood pressure levels to determine the antihypertensive medication recommendation primarily based on the results of a diverse set of data from post-hoc randomized clinical trials, observational studies and computer simulation analyses [17, 18]. Large-scale RCTs focusing on the combination of CVD risk and blood pressure levels to determine antihypertensive medication recommendations should be further encouraged. In addition, using the pooled CVD risk equation is not fully popularized in clinical practice and is not feasible for evaluation in the primary health care service setting in China. We also have to consider the overuse of antihypertensive drugs based on the 2017 ACC/AHA guideline concerning recommendation for antihypertensive medication. Finally, we should consider the recommendation carefully and conduct some studies in diverse populations to focus on the necessity of antihypertensive medication use in China.

The control rate of hypertension is relatively low compared to that in Western countries, although it has increased in recent years [4, 5]. According to the 2017 ACC/AHA hypertension guideline, the percentage of individuals with blood pressure above the goal increased from 39.0 to 53.4% in the US and increased from 53.3 to 88.8% in China among adults taking antihypertensive medications. In fact, we temporarily suspend the definition of this target and whether it is suitable for Chinese adults. The current situation of high prevalence and low control rate of hypertension in China is unsatisfactory. The benefits of antihypertensive medications for the reduction of CVD events are commonly accepted. Therefore, whether referring to the 2017 ACC/AHA hypertension guideline or the 2010 Chinese hypertension guideline, it is important and necessary to further improve the control rate of hypertension and provide more positive treatment and management for prevalent hypertension. Our results can provide a consult to government and public health agency for seeing the reality of hypertension in China and helping promote public health programs to increase hypertension awareness. Further management strategies for controlling hypertension and reducing the burden of CVD events are essential to promote the Goal of Healthy China 2030.

The present study has strength in that the study population is nationally representative. However, some limitations should also be considered in light of these results. First, blood pressure was measured only once, which may result in the misclassification of blood pressure. Second, we did not have sufficient information from laboratory measurements such as cholesterol, serum glucose, and family history of CVD, to calculate CVD risk, which may have helped us to evaluate the exact impact of the recommended antihypertensive medication on the Chinese population.

## Conclusion

The current analysis demonstrates that the 2017 ACC/AHA hypertension guideline have a potential impact on the prevalence of hypertension, the prevalence of the recommendation for antihypertensive medication and control rate in the Chinese hypertension population. The prevalence of hypertension will experience a substantial double increase, and the percentage of individuals recommended to take antihypertensive medications will have a small increase (2.7%). In addition, the control rate of hypertension will decrease sharply from 46.7 to 11.2% among adults taking antihypertensive medications. It is important to determine how to address the 2017 ACC/AHA hypertension guideline? We should pay more attention to the positive impact of the 2017 ACC/AHA guideline and perform some cost-effectiveness analyses regarding the implementation of the guideline. Regardless, we should positively consider the control and management of hypertension, based on the real policies of China to further reduce the burden of CVD in China.

## Data Availability

The datasets generated and/or analyzed during the current study are available in the China Health Nutrition Survey repository. Web link to datasets: https://www.cpc.unc.edu/projects/china.

## Abbreviations

ACC/AHA: American College of Cardiology / American Heart Association
CHNS: China health and nutrition survey
BP: Blood pressure
CI: Confidence interval
JNC7: Seventh report of the joint National Committee
NHANES: National Health and nutrition examination survey
SBP: Systolic blood pressure
DBP: Diastolic blood pressure
CAPM: Chinese academy of preventive medicine
CVD: Cardiovascular disease
SD: Standard deviation
IQR: Interquartile range
